# A Rare Case of Apple Peel Ileal Atresia With Coexisting Colonic Atresia: Surgical Management and Outcomes

**DOI:** 10.7759/cureus.86290

**Published:** 2025-06-18

**Authors:** Aleksandra I Sadecka, Aleksandra Jasinska

**Affiliations:** 1 Department of Pediatric Surgery, Medical University of Warsaw, Warsaw, POL

**Keywords:** apple peel ileal atresia, congenital colonic atresia, congenital ileal atresia, neonatal surgery, neonate

## Abstract

Ileal atresia is one of the most frequent causes of congenital intestinal obstruction. Type IIIb, also known as apple peel atresia, is the least common subtype. Colonic atresia is a rare anomaly that can also cause neonatal intestinal obstruction. Both can coexist with other atypical malformations, further complicating diagnosis. The surgical management of these two gastrointestinal malformations differs between centers, and no standardized procedural guidelines currently exist. We present a case of a neonate born with apple peel ileal atresia coexisting with transverse colonic atresia, both successfully treated with primary anastomoses. The postoperative course was uneventful, and the patient was discharged with full oral feeding and regular bowel movements.

## Introduction

Ileal atresia is one of the most common causes of congenital intestinal obstruction, with an occurrence rate of 0.7 per 10,000 live births. Type IIIb (apple peel atresia) is the least common subtype, accounting for approximately 7% of cases [1,2]. This congenital anomaly may be associated with other structural defects, such as gastroschisis, omphalocele, congenital heart disease, craniofacial deformities, gallbladder agenesis, and congenital diaphragmatic hernia. Additionally, chromosomal or genetic syndrome, such as Down syndrome, is diagnosed in 3.8% of affected patients [1-4].

Colonic atresia is a rare anomaly, with an incidence rate of 4.2 per 10,000 live births [5]. Associated congenital anomalies include omphalocele, gastroschisis, anorectal malformations, and cardiac anomalies [6,7].

The surgical management of these two gastrointestinal malformations differs between centers, and no standardized procedural guidelines currently exist. Coexisting ileal apple peel and colonic atresia is a rare and infrequently reported anomaly.

## Case presentation

**Figure 1:**
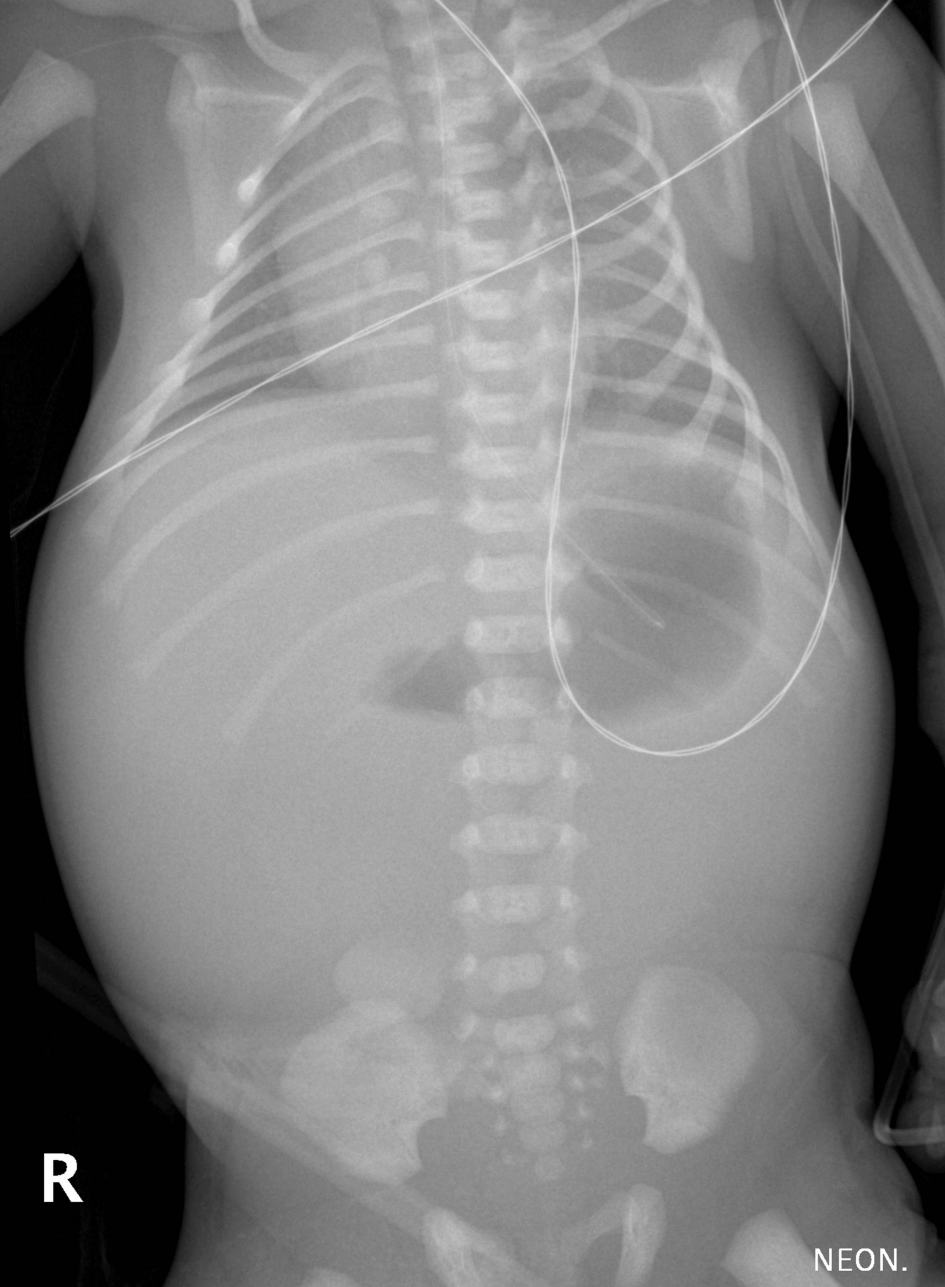
An abdominal X-ray showing a gas-filled gastric bubble and an air-fluid level in the duodenal bulb, with no gas visible in the distal intestine

A one-day-old neonate was admitted to the hospital due to a suspected gastrointestinal obstruction. The patient’s prenatal history included maternal polyhydramnios and dilated intestinal loops. He was born at 34 weeks of gestation in good condition, with an Apgar score of 10 and a birth weight of 2,560 g. On admission, he was hemodynamically stable and showed no signs of fluid or electrolyte imbalance. The patient presented with bilious vomiting and abdominal distension. Abdominal X-ray revealed a gas-filled gastric bubble and an air-fluid level in the duodenal bulb, with no gas visible in the distal intestine (Figure 1).

**Figure 2:**
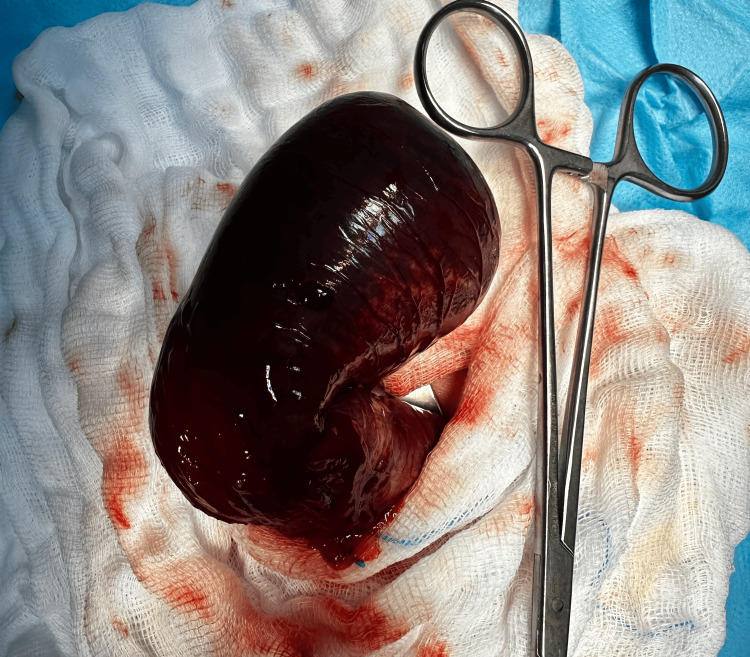
Enlarged, ischemic proximal jejunum

**Figure 3:**
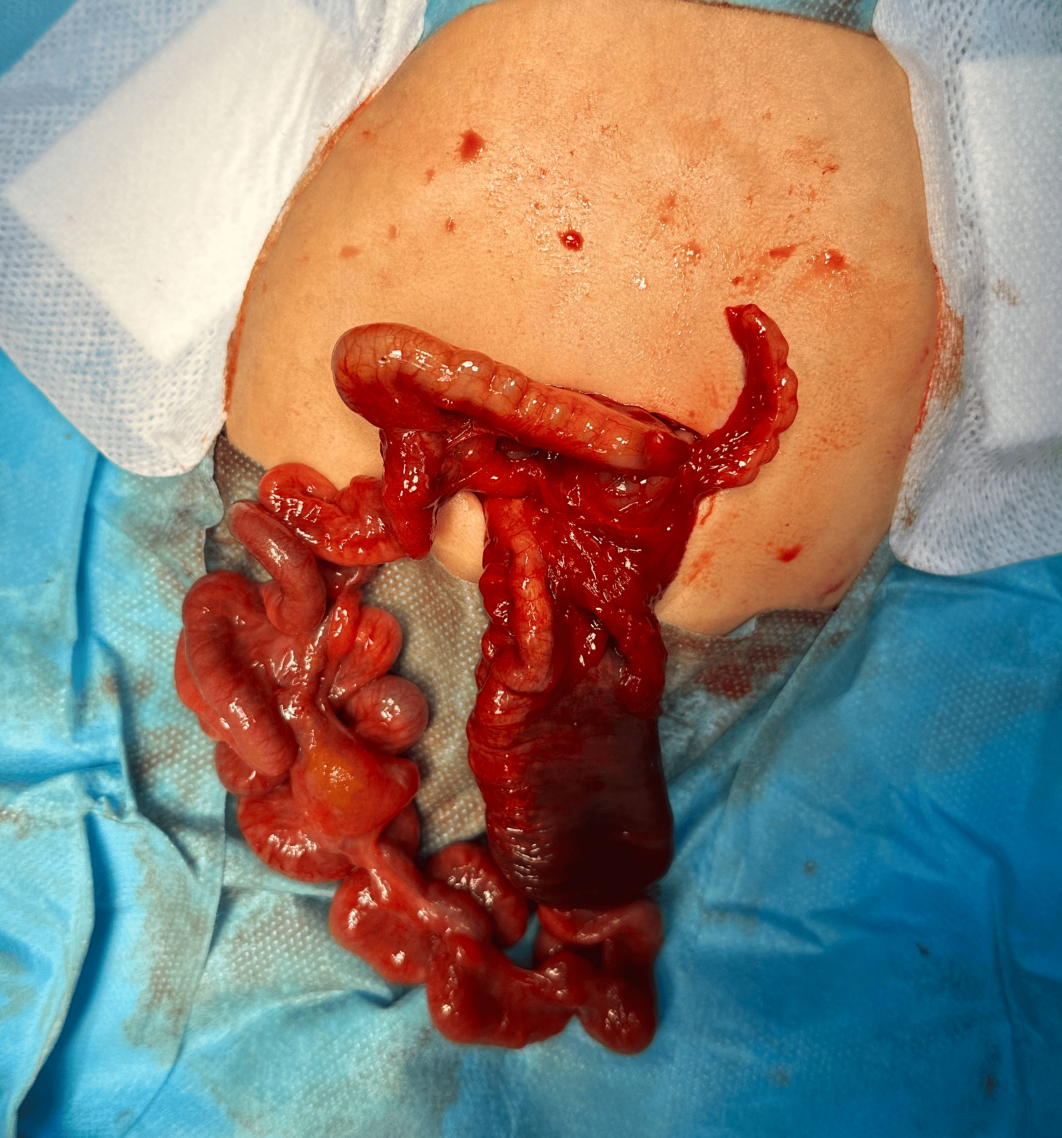
Apple peel ileal atresia and transverse colon atresia

The patient was deemed eligible for a surgical intervention. An upper transverse laparotomy was performed via a supraumbilical incision. Intraoperatively, apple peel ileal atresia and transverse colon atresia were identified. Surgical management involved a stapled resection of approximately 4 cm of the massively enlarged, ischemic proximal jejunum (Figure 2), followed by side-to-end jejunoileal anastomosis, and an end-to-end colonic anastomosis (Figure 3). Both anastomoses were performed in a single layer using absorbable, semi-continuous sutures. The ileocecal valve was preserved, and approximately 50 cm of small intestine was left in continuity.

Feeding was initiated on the third postoperative day. The postoperative course was uneventful. The patient was discharged with full oral feeding and regular bowel movements, with no signs of short bowel syndrome. At six-month follow-up, no complications have been reported.

## Discussion

Prenatal diagnosis of intestinal obstruction is typically based on ultrasound imaging, with fetal MRI in some cases. Radiologic findings include maternal polyhydramnios, dilated fetal bowel loops, “double bubble” or “triple bubble” sign [8,9]. However, the absence of detectable anomalies on prenatal assessment does not exclude the possibility of postnatal gastrointestinal obstruction [10].

Clinical symptoms of intestinal obstruction in neonates include abdominal distension, emesis (in patients with high gastrointestinal obstruction, usually bilious emesis or excessive gastric residuals), absence of meconium passage, and sometimes jaundice [1,10]. Postnatal radiological diagnosis of the intestinal obstruction in neonates involves ultrasound examination and abdominal X-ray. Typical signs include dilated bowel loops and air fluid levels [1]. In some cases, contrast fluoroscopy of the gastrointestinal tract is used to confirm complete gastrointestinal obstruction and may reveal a microcolon [1,10]. In our patient, the typical clinical presentation and radiologic findings on abdominal ultrasound and X-ray were sufficient to proceed with surgical intervention.

Management of ileal obstruction involves exploratory laparotomy or, in some cases, laparoscopy. Various surgical approaches for managing type IIIB ileal atresia (apple peel) have been reported in the literature. Rashwan et al. [11] presented a case series of 12 neonates treated for apple-peel atresia. All patients underwent primary anastomosis with the insertion of a T-tube into the proximal ileal segment. The authors reported that spontaneous closure occurred after T-tube removal in all cases, and no additional surgeries were required. One patient died due to sepsis, while the remaining neonates were discharged after a mean hospital stay of 22 days [11]. Kurdi et al. [12] compared antimesenteric sleeve enteroplasty with end-to-side primary anastomosis in 57 patients with ileal atresia. This study revealed several advantages of enteroplasty, including early initiation and maintenance of oral feeding, and improved intestinal transit. Significant postoperative complications were reported in three patients in the enteroplasty group and in five patients in the primary anastomosis group [12]. Mangray et al. reported on 34 patients treated for ileal atresia. Neonates underwent primary end-to-end anastomosis, enteroplasty with anastomosis, or creation of the Bishop-Koop stoma or ileostomy. In 11 patients, resection of the proximal bowel was necessary due to ischaemia or bulbous dilatation. Re-operations were required in 12 cases due to anastomosis leak, adhesive obstruction, volvulus, anastomosis stricture, and, in two cases, no abnormalities were found during re-laparotomy. The reported mortality rate was 70.5%, mainly due to sepsis [13]. This significantly differs from our local outcomes; therefore, the high complication rate presented by the authors should not be directly extrapolated to the surgical risk in our patient population.

The surgical approach in patients with colonic atresia also varies and depends on associated conditions, timing of the diagnosis, and local protocols. El-Asmar et al. [6] reported a series of 12 patients with colonic atresia. Surgical management in most cases consisted of creating a temporary Mikulicz stoma, while, in others, primary anastomosis with or without ileostomy was performed. Mortality and morbidity in this cohort were mainly attributed to additional congenital conditions and postoperative complications such as stoma-induced electrolyte imbalance and sepsis. The authors did not identify any of these approaches as definitively superior, but highlighted the importance of histopathological exclusion of aganglionosis in these patients [6]. Saha et al. [7] described a single-center experience involving 11 patients with colonic atresia. Surgical strategies included primary resection and anastomosis, colostomy, or ileocolostomy. The authors concluded that a staged approach with initial colostomy is preferable and emphasized the importance of rectal and distal colonic biopsy to rule out aganglionosis. Hirschsprung disease has been reported to coexist with gastrointestinal atresia in approximately 0.9% of cases [7,14]. To our knowledge, coexisting apple peel atresia and colonic atresia have not been previously reported. However, Yardley et al. [15] reported a case of a patient with type IV ileal atresia coexisting with colonic atresia. The authors decided to perform multiple end-to-end anastomoses over a transanastomotic tube, which wes exteriorized through the anus. No complications were reported, and the patient achieved full enteral feeding by postoperative day 25 [15].

## Conclusions

Surgical management of two coexisting rare congenital malformations is challenging. In cases of dilated proximal ileal atresia, the bowel wall is often significantly thickened, aperistaltic, and sometimes even ischemic. We conclude that resection of the affected segment and primary side-to-end ileal and end-to-end colonic anastomosis, without enteroplasty or stoma creation, may be beneficial, allowing for the preservation of maximal intestinal length. Moreover, performing a primary anastomosis allowed us to avoid the need for a second surgery and enabled early initiation of enteral feeding. Importantly, patients with congenital anomalies should be treated in specialized, multidisciplinary centers equipped with appropriate neonatal and surgical infrastructure. We believe that accurate prenatal diagnostics would facilitate maternal referral to a higher-level center before delivery, thus avoiding the need for neonatal transport within the first day of life.
